# Endophenotyping reveals differential phenotype-genotype correlations between myopia-associated polymorphisms and eye biometric parameters

**Published:** 2012-03-30

**Authors:** Jian Huan Chen, Haoyu Chen, Shulan Huang, Jianwei Lin, Yuqian Zheng, Mingliang Xie, Wenjie Lin, Chi Pui Pang, Mingzhi Zhang

**Affiliations:** 1 Joint Shantou International Eye Center, Shantou University & the Chinese University of Hong Kong, Shantou, China; 2 Department of Ophthalmology and Visual Sciences, the Chinese University of Hong Kong, Hong Kong, China; 3 Nan’ao People Hospital, Shantou, China

## Abstract

**Purpose:**

To investigate the association with ocular biometric parameters in myopia-associated single nucleotide polymorphisms (SNPs) of the gap junction protein delta 2 (*GJD2*), insulin-like growth factor-1 (*IGF1*) and hepatocyte growth factor (*HGF*) genes in two geographically different Chinese cohorts.

**Methods:**

In 814 unrelated Han Chinese individuals aged above 50 years including 362 inland residents and 432 island dwellers, comprehensive ophthalmic examinations were performed. Three SNPs, including *GJD2* rs634990, *IGF1* rs6214, and *HGF* rs3735520, were genotyped. Genetic association with ocular biometric parameters was analyzed in individual cohorts, using linear regression controlled for sex and age. Common associations shared by the two cohorts were revealed by meta-analysis.

**Results:**

Meta-analysis showed that *GJD2* rs634990 alone was not associated with any biometric parameters (adjusted p>0.645). The T allele of *IGF1* rs6214 was specifically associated with thicker lens (β±SE=0.055±0.022, adjusted p=0.034). The A allele of *HGF* rs3735520 was associated with longer vitreous chamber depth (β±SE=0.143±0.060, adjusted p=0.050). Significant interaction between *HGF* rs3735520 and *GJD2* rs634990 was found in association with axial length and vitreous chamber depth (adjusted p=0.003 and 0.033, respectively), and possibly with spherical error (adjusted p=0.056).

**Conclusions:**

Our endophenotyping analysis showed differential association between selected myopia-associated genes and ocular biometric parameters in our Chinese cohorts, which may underline substantial but diversified effects of these genes and their interaction on the development of eye structure and etiology of myopia.

## Introduction

Myopia is one of the most common causes of visual impairment [1-5]. It is estimated that about 33.1% of the USA population is affected by this disorder [5]. The prevalence of myopia in China has been reported to be even higher, and up to 80% of Chinese children can have myopia [6,7]. Severe myopia is often linked to clinical complications [8], even permanent visual loss [9]. Myopia is an ultimate manifestation resulting from changes of eye structure or compartment in the optical path, which consists of cornea, anterior chamber, lens, and vitreous chamber [10,11]. These biometric parameters and the myopia disorder itself have been shown to have large genetic predisposition, implicating that these genetic determinants of ocular parameters can possibly influence the risk to myopia by controlling ocular development [12-15].

Recently genome-wide association studies (GWAS) on quantitative traits have been successfully identified gene and variants associated with myopia. Variants at chromosome 15q14 and 15q25 have been reported to be associated with myopia and refractive error in two independent Caucasian GWAS [16,17]. Among these variants, single nucleotide polymorphisms (SNPs) of the gap junction protein delta 2 (*GJD2*) gene at 15q14 was reported to be more significantly associated with high myopia compared to SNPs at 15q25 in Japanese [18]. The *GJD2* gene encodes connexin 36, a 36 kDa protein, which is a member of the connexin gene family and is highly expressed in mouse and human retina [19]. The connexin family can possibly be involved in ocular development and various eye diseases [20]. The quantitative trait association of *GJD2* with refractive error thus remains to be investigated in Chinese. In addition to connexins, growth factors also play a substantial role in ocular development, and may influence biometric parameters [21]. The insulin-like growth factor 1 (*IGF1*) gene within the myopia 3 (high grade, autosomal dominant, *MYP3*) locus [22], has been reported to be associated with myopia in Caucasians [23]. Expression of *IGF1* mRNA in chicken ocular tissues can be affected by myopic or hyperopic defocus [24]. Likewise, association of the hepatocyte growth factor gene (*HGF*) with myopia has also been reported in Chinese [25] and Caucasians [26]. But quantitative trait association of both growth factor genes with ocular biometric parameters has not yet been studied. It remains to be investigated whether these three myopia-associated genes affect ocular development.

In the current study we investigated the association of three myopia-associated genes, *GJD2*, *IGF1*, and *HGF*, and their interaction with eye biometric parameters in two Chinese cohorts. Our findings may suggest the substantial role of these genetic polymorphisms in shaping eye structure and development of myopia.

## Methods

### Patient recruitment and clinical information

This study was approved by the Ethics Committee of Joint Shantou International Eye Center, Shantou, China and was conducted in accordance with the Declaration of Helsinki. Written consent was obtained from each participating subject after explanation of the nature of the study.

The study subjects included 814 unrelated Han Chinese living all their lives in two geographical different regions in Southeastern China: 362 unrelated inland residents aged 50 and older, recruited from senile cataract surgical patients at Joint Shantou International Eye Center in Shantou (STM), and 432 unrelated local dwellers aged 50 and older, recruited from Nan’ao Island (NAI). The eyes with the following conditions were excluded: any history or symptom of Marfan’s syndrome, ocular trauma, ocular surgery, macular epiretinal membrane, macular edema, macular hemorrhage, glaucoma, or retinal detachment. Eye biometric parameters were documented for all study subjects.

All participants received comprehensive ophthalmic examination including best-corrected visual acuity, slit-lamp biomicroscopy of anterior segment and retina with mydriasis, Refractive parameters including astigmatism, corneal curvature, spherical error, and cylindrical error were measured by auto refractometer (RK-F1 Refractometer/Keratometer; Canon, Inc., Tochigi, Japan). Spherical equivalent was calculated as spherical error plus half of cylindrical error. Astigmatism was calculated as the difference between anterior and posterior cornea curvatures, and corneal curvature was calculated as the mean of the two. Axial length, anterior chamber depth, lens thickness, and vitreous chamber depth measured by A-scan ultrasound biometry (ODM 2200; Tianjin Maida Medical Technology Co., Ltd., Tianjin, China). The central corneal thickness (CCT) was measured ultrasonically (IOPac 20Mhz Pachymeter; Heidelberg Engineering, Heidelberg, Germany). Eyes with prior surgical history or low data quality were excluded. For 557 individuals with bilateral data available, the means of biometric parameters was used to represent the data from both eyes. For 257 individuals with data from OD or OS data unavailable, data from the contralateral eye were used. Peripheral blood was collected from all participants, and genomic DNA was extracted using the Qiamp Blood Kit (Qiagen, Hilden, Germany).

### SNP genotyping

Three SNPs including rs634990 in *GJD2*, rs6214 in *IGF-1*, and rs3735520 in *HGF* were genotyped by *Taqman* SNP Genotyping assay (Applied Biosystems, Inc. [ABI], Foster City, CA) following the protocol suggested by the manufacturer.

### Statistical analysis

Hardy–Weinberg Test of each SNP was conducted using Haploview version 4.2 [27]. Gender difference between the two cohorts was compared using χ^2^ tests, and age and biometric parameters were compared using non-parametric Mann–Whitney U test. Linear regression implemented by the R statistical language version 2.12.1 was used to analyze quantitative trait association for each individual cohort separately, controlling gender and age as described in previous studies [28,29]. The additive genetic model was used, assuming a trend per copy of the minor allele. Homozygous major, heterozygous, and homozygous minor genotypes were coded as 0, 1, and 2 in the regression. Effect size±standard error (β±SE) of per copy of minor allele was calculated for each SNP accordingly. With the homozygous major genotypes as the reference (0 × β), heterozygous and homozygous minor genotypes were estimated to account for 1×β and 2×β changes of biometric parameters, respectively. To identify common associations shared by the two Chinese cohorts, meta-analysis was further performed using fixed-effect models and inverse variance weighting methods implemented by METAL [30]. Bonferroni correction for multiple comparisons was applied to adjust meta-analysis p-values.

## Results

### Demographic and clinical data

**Figure 1:**
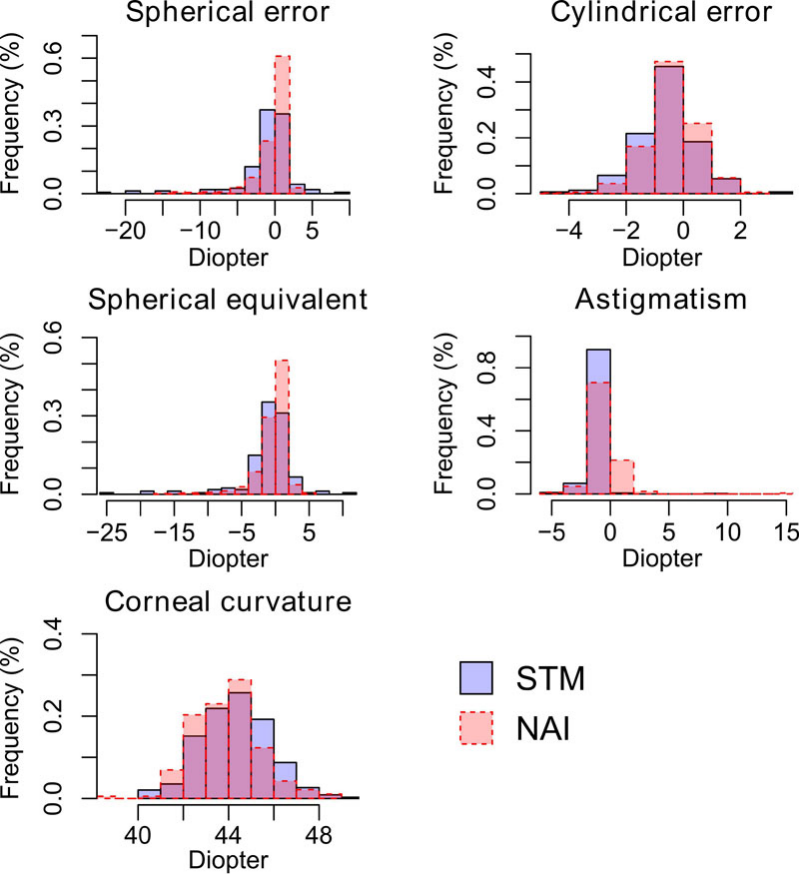
Distribution of refractive parameters in both the inland (STM) and island (NAI) cohorts. Histogram of the STM cohort is shown in light blue and that of the NAI cohort is in semitransparent red.

**Figure 2:**
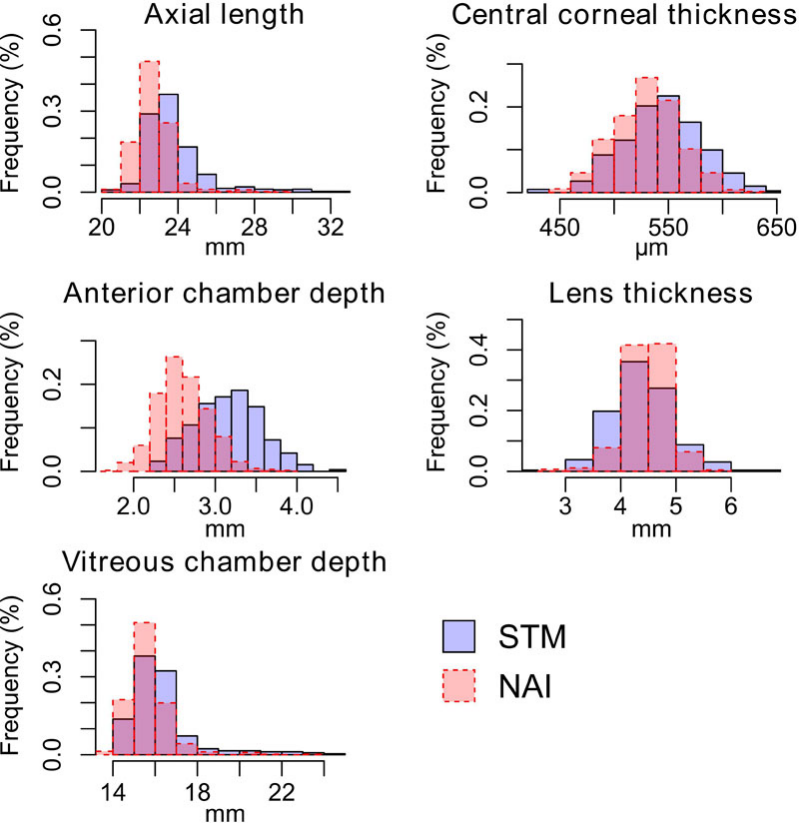
Distribution of axial ocular dimensions in both both the inland (STM) and island (NAI) cohorts. Histogram of the STM cohort is shown in light blue and that of the NAI cohort is in semitransparent red.

The distribution of refractive parameters and axial ocular dimensions in both STM and NAI cohorts were shown in Figure 1 and Figure 2. As Summarized in Table 1, comparison between the two Chinese cohorts showed significantly lower female proportion and older mean age in STM. It also revealed significant difference in both refractive parameters and axial ocular dimensions (all Mann–Whitney U test p<0.044). The STM cohort was in average more myopic with longer mean axial length, anterior chamber depth, and vitreous chamber depth, and thicker central cornea.

**Table 1:** Demographic information and clinical features of the study subjects.

**Category**	**STM**	**NAI**	p*
**Gender**			
Male	139	119	< 0.001
Female	223	333	
**Age (Year)**			
Mean	71.8	62.3	< 0.001
(SD)	(7.9)	(9.2)	
**Spherical error (D)**			
Mean	−0.9	−0.3	0.001
(SD)	(3.8)	(2.3)	
**Cylindrical error (D)**			
Mean	−0.4	−0.3	0.044
(SD)	(1.1)	(0.9)	
**Spherical equivalent (D)**			
Mean	−1.2	−0.4	< 0.001
(SD)	(4.1)	(2.5)	
**Astigmatism (D)**			
Mean	−1.0	−0.7	< 0.001
(SD)	(0.9)	(1.6)	
**Curvature (D)**			
Mean	44.3	44.0	0.006
(SD)	(1.5)	(1.5)	
**Axial length (mm)**			
Mean	23.8	22.7	< 0.001
(SD)	(1.7)	(1.1)	
**Central corneal thickness (µm)**			
Mean	544.6	531.2	< 0.001
(SD)	(46.9)	(30.7)	
**Anterior chamber depth (mm)**			
Mean	3.2	2.6	< 0.001
(SD)	(0.4)	(0.3)	
**Lens thickness (mm)**			
Mean	4.4	4.5	0.004
(SD)	(0.6)	(0.4)	
**Vitreous chamber depth (mm)**			
Mean	16.2	15.7	< 0.001
(SD)	(1.6)	(1.0)	

* χ^2^ tests were used for gender ratio comparison, and Mann–Whitney U tests were used for comparison of age and biometric parameter between the two cohorts.

### Single gene association

None of the SNPs genotyped in the current study showed deviation from Hardy–Weinberg Equilibrium in either STM or NAI cohort (all p-value >0.05), and thus were subsequently included in further association study. The three SNPs showed similar minor allele frequencies between the two Chinese cohorts (42.6%–49.0%, Table 2).

**Table 2:** The frequencies of alleles and genotypes in the inland and island cohorts.

** **	** **	**Allele statistics**						**Genotype statistics**					
** **	** **	** **	**Frequency (%)**				** **	** **	**Frequency (%)**				** **
**Gene**	**SNP**	**Allele**	**STM**		**NAI**		p*	**Genotype**	**STM**		**NAI**		p*
*GJD2*	rs634990	T	376	(52.4)	495	(55.5)	0.230	TT	103	(28.7)	132	(29.6)	0.170
** **	** **	C	342	(47.6)	397	(44.5)	** **	CT	170	(47.4)	231	(51.8)	** **
** **	** **	** **	** **	** **	** **	** **	** **	CC	86	(24.0)	83	(18.6)	** **
*IGF1*	rs6214	C	378	(52.6)	455	(51.0)	0.546	CC	109	(30.4)	122	(27.4)	0.620
** **	** **	T	340	(47.4)	437	(49.0)	** **	CT	160	(44.6)	211	(47.3)	** **
** **	** **	** **	** **	** **	** **	** **	** **	TT	90	(25.1)	113	(25.3)	** **
*HGF*	rs3735520	G	408	(57.1)	510	(57.4)	0.950	GG	118	(33.1)	145	(32.7)	0.910
** **	** **	A	306	(42.9)	378	(42.6)	** **	AG	172	(48.2)	220	(49.5)	** **
** **	** **	** **	** **	** **	** **	** **	** **	AA	67	(18.8)	79	(17.8)	** **

*p-values derived from χ^2^ tests of allele or genotype frequencies.

Additive genetic models assuming a trend per copy of the minor allele were first used to test the association between biometric parameters and genotypes in each gene alone by using both eye data. As shown in Table 3 and Table 4, quantitative association analysis showed that *GJD2* rs634990 was association with central corneal thickness (β±SE=-9.386±3.517 µm, p=0.008) in cohort STM. The association was not consistent in cohort NAI (β±SE=0.819±2.131 µm, p=0.701), and became insignificant in meta-analysis (adjusted p=0.965). *GJD2* rs634990 was not associated with any other refractive parameter or ocular dimension (all meta-analysis adjusted p>0.645).

**Table 3:** Association of *GJD2* rs634990, *IGF1* rs6214, and *HGF* rs3735520 with refractive parameters.

** **	** **	**STM**			**NAI**			**Meta-analysis**			
**Gene**	**SNP**	**β**	**SEM**	**p**	**β**	**SEM**	**p**	**β**	**SEM**	**p**	**Adjusted p**
**Spherical error**											
*GJD2*	rs634990	−0.466	0.404	0.251	0.089	0.172	0.604	0.004	0.158	0.981	1.000
*IGF1*	rs6214	−0.566	0.412	0.171	0.048	0.160	0.766	−0.032	0.149	0.828	0.995
*HGF*	rs3735520	−1.028	0.402	0.011	−0.175	0.168	0.298	−0.302	0.155	0.052	0.147
**Cylindrical error**											
*GJD2*	rs634990	−0.092	0.117	0.433	−0.011	0.072	0.882	−0.033	0.061	0.588	0.930
*IGF1*	rs6214	−0.181	0.120	0.132	0.069	0.066	0.297	0.011	0.058	0.850	0.997
*HGF*	rs3735520	−0.219	0.117	0.064	0.034	0.069	0.619	−0.031	0.059	0.599	0.935
**Spherical equivalent**											
*GJD2*	rs634990	−0.512	0.433	0.239	0.093	0.188	0.623	−0.003	0.172	0.986	1.000
*IGF1*	rs6214	−0.657	0.441	0.138	0.055	0.174	0.753	−0.041	0.162	0.801	0.992
*HGF*	rs3735520	−1.137	0.430	0.009	−0.153	0.182	0.402	−0.302	0.168	0.071	0.198
**Astigmatism**											
*GJD2*	rs634990	−0.017	0.069	0.809	−0.087	0.168	0.605	−0.027	0.064	0.671	0.964
*IGF1*	rs6214	−0.031	0.068	0.652	0.088	0.163	0.59	−0.013	0.063	0.831	0.995
*HGF*	rs3735520	0.008	0.071	0.906	−0.256	0.164	0.12	−0.034	0.065	0.605	0.939
**Corneal curvature**											
*GJD2*	rs634990	0.085	0.110	0.439	−0.088	0.159	0.579	0.029	0.091	0.749	0.984
*IGF1*	rs6214	0.234	0.107	0.029	0.074	0.154	0.629	0.182	0.088	0.038	0.111
*HGF*	rs3735520	−0.128	0.113	0.256	0.192	0.156	0.222	−0.018	0.092	0.845	0.996

**Table 4:** Association of *GJD2* rs634990, *IGF1* rs6214, and *HGF* rs3735520 with axial ocular dimensions.

** **	** **	**STM**			**NAI**			**Meta-analysis**			
**Gene**	**SNP**	**β**	**SEM**	p*	**β**	**SEM**	**p**	**β**	**SEM**	**p**	**Adjusted p**
**Axial length**											
*GJD2*	rs634990	0.052	0.124	0.678	−0.042	0.070	0.544	−0.019	0.061	0.752	0.985
*IGF1*	rs6214	0.091	0.121	0.452	−0.009	0.066	0.896	0.014	0.058	0.810	0.993
*HGF*	rs3735520	0.347	0.126	0.006	0.015	0.069	0.831	0.092	0.061	0.130	0.342
**Central corneal thickness**											
*GJD2*	rs634990	−9.386	3.517	0.008	0.819	2.131	0.701	−1.922	1.823	0.292	0.645
*IGF1*	rs6214	−3.054	3.452	0.377	−1.454	2.015	0.471	−1.861	1.740	0.285	0.634
*HGF*	rs3735520	−2.180	3.674	0.553	2.245	2.104	0.287	1.152	1.826	0.528	0.895
**Anterior chamber depth**											
*GJD2*	rs634990	0.005	0.030	0.865	−0.024	0.022	0.276	−0.014	0.018	0.435	0.819
*IGF1*	rs6214	−0.011	0.029	0.709	−0.008	0.020	0.699	−0.009	0.017	0.586	0.929
*HGF*	rs3735520	0.002	0.031	0.937	−0.013	0.021	0.554	−0.008	0.017	0.634	0.951
**Lens thickness**											
*GJD2*	rs634990	0.018	0.042	0.674	−0.002	0.028	0.935	0.004	0.023	0.859	0.997
*IGF1*	rs6214	0.049	0.041	0.240	0.058	0.026	0.027	0.055	0.022	0.012	**0.034**
*HGF*	rs3735520	0.018	0.044	0.687	−0.044	0.027	0.113	−0.027	0.023	0.240	0.561
**Vitreous chamber depth**											
*GJD2*	rs634990	−0.006	0.117	0.963	−0.030	0.070	0.669	−0.024	0.060	0.694	0.971
*IGF1*	rs6214	0.021	0.114	0.854	−0.044	0.066	0.506	−0.028	0.057	0.628	0.948
*HGF*	rs3735520	0.348	0.120	0.004	0.075	0.069	0.277	0.143	0.060	0.017	**0.050**

For *IGF1* rs6214, association between its minor allele T and corneal curvature was detected in STM (β±SE=0.23±0.11 D, p=0.029, Table 3). The association was insignificant in cohort NAI (p=0.629) and meta-analysis (adjusted p=0.111). The same allele T of *IGF1* rs6214 showed a trend of association with longer lens thickness in both STM and NAI cohorts (β±SE=0.049±0.041 mm, p=0.240; and β±SE=0.06±0.03 mm, p=0.027, respectively, Table 4). The association remained significant in meta-analysis (β±SE=0.055±0.022 mm, adjusted p=0.034). No significant effects of *IGF1* rs6214 was found on any other biometric parameters (all p>0.05).

For *HGF* rs3735520, its minor allele A showed effects of negative spherical error and spherical equivalent, and longer axial length in STM (β±SE=-1.03±0.40 D, p=0.011; β±SE=-1.14±0.43 D, p=0.009 and β±SE=0.35±0.13 mm, p=0.006, respectively, Table 3). These associations did not reach statistical significance in cohort NAI (p=0.298, 0.402 and, 0.831, respectively). Meta-analysis did not found significance in these associations (adjusted p=0.147, 0.198, and 0.342, respectively). The same A allele of rs3735520 A showed a trend of association with longer vitreous chamber depth both in STM (β±SE=0.46±0.14 mm, p=0.001), and NAI (β±SE=0.075±0.069 mm, p=0.277). And the association was marginally significant in meta-analysis (β±SE=0.148±0.062 mm, adjusted p=0.050). No association of *HGF* rs3735520 was found with any other biometric parameters (all p>0.05).

The same analysis was also performed using one eye data (Appendix 1 and Appendix 2), and the findings were comparable to the results above using both eye data.

### Gene-gene interaction

As shown in Table 5 and Table 6, meta-analysis of two-locus interaction was performed for the association of the three genes with ocular biometric parameters. By using meta-analysis, significant interaction between *GJD2* rs634990 and *HGF* rs3735520 was revealed in association with axial length and vitreous chamber depth (β±SE=-0.298±0.090, adjusted p=0.003 and β±SE=-0.223±0.088, adjusted p=0.033, respectively). With the interaction item included in the full linear regression model, *HGF* rs3735520 showed significant effects on axial length and vitreous chamber depth (β±SE=0.373±0.104, adjusted p=0.001 and β±SE=0.359±0.103, adjusted p=0.001, respectively), and *GJD2* rs634990 was associated with axial length (β±SE=0.231±0.096, adjusted p=0.049). Marginal significant interaction between *GJD2* rs634990 and *HGF* rs3735520 was also found in association with spherical error and spherical equivalent (β±SE=0.540±0.231, adjusted p=0.056 and β±SE=0.559±0.250, adjusted p=0.075, respectively). When the interaction item included in the full linear regression model, *HGF* rs3735520 showed significant effects on axial length and vitreous chamber depth (β±SE=-0.804±0.226, adjusted p=0.006 and β±SE=-0.828±0.285, adjusted p=0.011, respectively). No significant interaction of *IGF1* rs6214 with either of the other two genes was found in meta-analysis.

**Table 5:** Two-locus interaction analysis in association with refractive parameters.

** **	** **	**STM**			**NAI**			**Meta-analysis**				
**Gene 1**	**Gene 2**	**β**	**SEM**	**p**	**β**	**SEM**	**p**	**β**	**SEM**	**p**	**Adjusted p**	
**Spherical error**												
*GJD2*	*IGF1*	0.610	0.564	0.281	−0.446	0.235	0.058	−0.290	0.217	0.182	0.452	** **
*GJD2*	*HGF*	0.588	0.531	0.269	0.529	0.256	0.039	0.540	0.231	0.019	**0.056**	*
*IGF1*	*HGF*	0.380	0.536	0.479	0.296	0.226	0.191	0.309	0.208	0.138	0.360	** **
**Cylindrical errors**												
*GJD2*	*IGF1*	−0.107	0.164	0.515	−0.051	0.098	0.603	−0.066	0.084	0.435	0.819	** **
*GJD2*	*HGF*	0.150	0.156	0.339	−0.009	0.106	0.935	0.041	0.088	0.638	0.953	** **
*IGF1*	*HGF*	0.132	0.157	0.402	0.033	0.093	0.721	0.059	0.080	0.463	0.845	** **
**Spherical equivalent**												
*GJD2*	*IGF1*	0.557	0.605	0.359	−0.512	0.257	0.047	−0.349	0.237	0.141	0.365	** **
*GJD2*	*HGF*	0.663	0.568	0.245	0.534	0.279	0.057	0.559	0.250	0.026	**0.075**	**
*IGF1*	*HGF*	0.446	0.573	0.437	0.314	0.246	0.203	0.335	0.226	0.139	0.362	** **
**Astigmatism**												
*GJD2*	*IGF1*	0.082	0.095	0.387	0.027	0.239	0.911	0.075	0.088	0.399	0.783	** **
*GJD2*	*HGF*	−0.010	0.097	0.918	−0.211	0.252	0.403	−0.036	0.091	0.691	0.971	** **
*IGF1*	*HGF*	0.040	0.095	0.677	0.123	0.228	0.592	0.052	0.088	0.551	0.910	** **
**Corneal curvature**												
*GJD2*	*IGF1*	−0.184	0.149	0.217	−0.236	0.225	0.296	−0.200	0.124	0.108	0.290	** **
*GJD2*	*HGF*	0.239	0.152	0.116	−0.013	0.241	0.957	0.167	0.129	0.193	0.475	** **
*IGF1*	*HGF*	0.082	0.149	0.584	−0.312	0.216	0.151	−0.045	0.123	0.714	0.976	** **

*After the interaction item included in the regression, β±SEM were −0.473±0.250 (adjusted p=0.165) and −0.804±0.226 (adjusted p=0.006) for *GJD2* rs634990 and *HGF* rs3735520, respectively. **After the interaction item included in the regression, β±SEM were −0.501±0.273 (adjusted p=0.186) and −0.828±0.285 (adjusted p=0.011) for *GJD2* rs634990 and *HGF* rs3735520, respectively.

**Table 6:** Two-locus interaction analysis in association with axial ocular dimensions.

** **	** **	**STM**			**NAI**			**Meta-analysis**				
**Gene 1**	**Gene 2**	**β**	**SEM**	**p**	**β**	**SEM**	**p**	**β**	**SEM**	**p**	**Adjusted p**	
**Axial length**												
*GJD2*	*IGF1*	−0.097	0.170	0.570	0.224	0.098	0.022	0.144	0.085	0.090	0.246	** **
*GJD2*	*HGF*	−0.243	0.171	0.157	−0.318	0.105	0.003	−0.298	0.090	0.001	**0.003**	*
*IGF1*	*HGF*	−0.111	0.167	0.505	−0.124	0.093	0.186	−0.121	0.081	0.137	0.357	** **
**Central corneal thickness**												
*GJD2*	*IGF1*	8.596	4.725	0.070	−5.621	3.009	0.062	−1.519	2.538	0.550	0.909	** **
*GJD2*	*HGF*	4.668	4.888	0.340	0.624	3.218	0.846	1.847	2.688	0.492	0.869	** **
*IGF1*	*HGF*	−0.160	4.857	0.974	5.192	2.833	0.068	3.833	2.447	0.117	0.312	** **
**Anterior chamber depth**												
*GJD2*	*IGF1*	0.012	0.041	0.776	−0.029	0.030	0.334	−0.015	0.024	0.544	0.905	** **
*GJD2*	*HGF*	−0.009	0.042	0.824	−0.032	0.033	0.329	−0.023	0.026	0.371	0.751	** **
*IGF1*	*HGF*	−0.093	0.041	0.024	−0.018	0.029	0.533	−0.043	0.024	0.069	0.194	** **
**Lens thickness**												
*GJD2*	*IGF1*	0.033	0.057	0.559	0.002	0.039	0.955	0.012	0.032	0.712	0.976	** **
*GJD2*	*HGF*	−0.043	0.059	0.458	−0.046	0.042	0.276	−0.045	0.034	0.189	0.466	** **
*IGF1*	*HGF*	0.097	0.058	0.093	0.014	0.037	0.694	0.038	0.031	0.223	0.531	** **
**Vitreous chamber depth**												
*GJD2*	*IGF1*	−0.144	0.159	0.366	0.260	0.098	0.008	0.149	0.083	0.075	0.207	** **
*GJD2*	*HGF*	−0.170	0.161	0.292	−0.246	0.105	0.020	−0.223	0.088	0.011	**0.033**	**
*IGF1*	*HGF*	−0.091	0.158	0.566	−0.127	0.093	0.175	−0.118	0.080	0.142	0.368	** **

*After the interaction item included in the logistic regression, β±SEM were 0.231±0.096 (adjusted p=0.049) and 0.373±0.104 (adjusted p=0.001) for *GJD2* rs634990 and *HGF* rs3735520, respectively. **After the interaction item included in the logistic regression, β±SEM were 0.165±0.095 (adjusted p=0.231) and 0.359±0.103 (adjusted p=0.001) for *GJD2* rs634990 and *HGF* rs3735520, respectively.

## Discussion

In the current study involving two geographically different Chinese cohorts, our results showed suggestive association of *IGF1* with lens thickness, and *HGF* with vitreous chamber depth. Hence our findings provided new insight into the roles of these myopia-associated genes in the development of different eye components in our Chinese cohorts and possibly in etiology of related eye diseases such as myopia.

Ocular development and myopia can be shaped by genetic and environmental factors [31,32]. In the current study, dramatic differences in baselines of ocular biometric parameters were found between an inland cohort STM and an island cohort NAI. Such difference could be due to lower female proportion and older mean age in STM. Moreover, it could be due to variable environmental exposure and lifestyle between the two. In contrast, the two Chinese cohorts have close genetic background in the three genes investigated in the current study. In spite of such difference in trait baselines between the two cohorts, common genetic correlation with ocular biometric parameters could be detected within each individual cohort, and further confirmed by a meta-analysis approach. These findings suggested that intrinsic genetic factors contributed to variations of ocular biometric parameters that could not be explained by environmental factors.

The quantitative trait association studies have been used to delineate genetic predisposition in these disease-related biometric parameters. Previously Solouki et al. [17] reported chromosome 15q14 spanning SNP rs634990 in *GJD2* to show genome-wide significance for association with refractive error in a Dutch population-based GWAS. The C allele of rs634990 was recently reported to confer risk to myopia in Japanese [18]. Although our cohorts showed similar minor allele frequency of rs634990 compared to the Hapmap Han Chinese data and the Japanese cohort, its association with spherical equivalent or other refractive parameters was not detected. The current findings might indicate ethnic difference in genetic predisposition of myopia between our Chinese cohorts and other reported populations. Moreover, our two-locus analysis results implicated that *GJD2* could play a role in myopia etiology by interacting with other myopia-associated genes in ocular development and association with biometric parameters.

The genotype frequencies of rs6214 in our Chinese cohorts were similar to the reported Han Chinese of Hapmap data. *IGF1* rs6214 was specifically associated with lens thickness in our Chinese cohorts. The minor allele of *IGF1* rs6214 was correlated with 0.07 mm increase of lens thickness in our meta-analysis Chinese cohort, which account for about 1.56 D change in refractive error according to previously reported approximately 0.045 mm/D change in lens thickness [33,34]. The lens of adult human accounts for about one third of the total refractive power in the eye [35]. Although correlation of *IGF1* with refractive error was not detected, the change of lens thickness could still potentially affect the ultimate refractive error. Previously, rs6214 was reported to be associated with both high myopia and myopia in an international Caucasian cohort [23]. Animal studies have implicated the role of the *IGF1* in lens development. *IGF1* has previously been reported to induce lens cell elongation and specialized crystallin gene expression in embryonic chicken eyes [36]. The association of *IGF1* with lens thickness but not with other ocular dimensions, constellated with the existing genetic association of *IGF1* with myopia, possibly implicated its specific role in refractive myopia.

In contrast to *IGF1*, *HGF* was specifically associated with axial length and vitreous chamber, but not lens thickness in our meta-analysis. The minor allele A was correlated with increased chamber depth in the meta-analysis. Axial length is one of the major determinants of refractive error, and accounts about 50% variance of spherical equivalent [15]. Vitreous chamber is the largest compartment in the eye, and its depth accounts for the largest proportion of axial length. These findings could explain the previous report of *HGF* as a high myopia-associated gene in the Chinese population [25]. Intriguingly, *HGF* exhibited significant interaction with another myopia-associated gene *GJD2*, which also contributed to the genetic association with axial length and vitreous chamber depth. Notably such interactive effects were also implicated in spherical error and spherical equivalent but not cylindrical error, and *HGF* was significantly associated with these two parameters when interaction was considered. *HGF* probably interact with *GJD2* to control the axial dimension and thus influence refractive parameters, which possibly explain its association with myopia. Axial length change has been estimated to be 0.35 mm/D in myopia [37], and thus the effect size of 0.377 mm per copy of rs3735520 minor allele was expected to account for approximately 1.07 D change of refractive error, which was close to the observed value of 0.828 D. And homozygous minor genotypes of rs3735520 with 2 copies of minor alleles could account for 1.656 D change of refractive error. Interestingly, in the Nepalese population *HGF* was recently reported to be associated with primary angle-closure glaucoma [38], in which patients were usually featured by shorter axial length and vitreous chamber depth. The SNP rs3735520 was associated with serum HGF level in normal individuals [39], suggesting its possible function link to gene expression. Taken together, *HGF* is probably involved in development of the posterior eye segment, and consequently in spherical error and axial myopia.

Myopia is characterized by major clinical features including negative refractive error and elongated eye axial length. However, both of these two features are ultimate phenotypes depending on various genes modulating the anatomic development of the eye. The differential correlation of myopia-associated genes with refractive error and axial ocular dimensions in the current study thus underlined the importance of endophenotyping in myopia genetics study. Firstly different genes or gene sets could be responsible for specific endophenotypes. Moreover, genes that controlled axial length could be of special interest. It has been reported that these genes account for approximately 50% of the variation in spherical equivalence [15]. Secondly, our data further pointed to a substantial role of interaction between these genes such as *HGF* and *GJD*, in genetic studies of myopia endophenotypes.

In the current study, we reported differential phenotype-genotype correlations between myopia-associated genes and eye biometric parameters in the Chinese population. *IGF1* was associated with lens thickness, *HGF* was associated with vitreous chamber depth, and the interaction between *HGF* and *GJD2* was associated with axial length, vitreous chamber depth and possibly spherical error. These findings provided new information in the diversified functional role of these susceptibility genes in myopia etiology and ocular development.

## Appendix 1.

Association of *GJD2* rs634990, *IGF1* rs6214, and *HGF* rs3735520 with refractive parameters using one eye data. To access the data, click or select the words “Appendix 1.” This will initiate the download of a compressed (pdf) archive that contains the file.

## Appendix 2.

Association of *GJD2* rs634990, *IGF1* rs6214, and *HGF* rs3735520 with axial ocular dimensions using one eye data. To access the data, click or select the words “Appendix 2.” This will initiate the download of a compressed (pdf) archive that contains the file.
